# Reconstruction after esophagectomy in patients with [partial] gastric resection. Case report and review of the literature of the use of remnant stomach

**DOI:** 10.1186/1477-7800-3-10

**Published:** 2006-04-26

**Authors:** Gianlorenzo Dionigi, Renzo Dionigi, Francesca Rovera, Luigi Boni, Giulio Carcano

**Affiliations:** 1 Department of Surgical Sciences, University of Insubria, Varese, Italy

## Abstract

**Background:**

Bowel reconstruction after subtotal esophagectomy represents a problem when a previous distal gastrectomy was performed: usually the colon or jejunum is used.

**Methods:**

In a 10 year period 126 patients with primary esophageal cancer underwent esophageal resection in our Department. Surgical procedures were 57% two-phase subtotal oesophagectomy, 23% transhiatal, 9% stripping, 10 three-phase total esophagectomy and 2 endoscopic resections.

**Results:**

In 112 patients alimentary tract reconstruction was achieved by means of esophago-gastric anastomosis. Reconstruction was performed using colon in 10 cases and jejunum in 2. We describe the technical aspects of esophagectomy and gastric reconstruction in a patient with previous antrectomy and Billroth II reconstruction. The procedure was performed via a combined laparotomy and thoracotomy with anastomosis at the level of the azygous vein using the remnant stomach.

**Conclusion:**

Few technical reports have been reported in literature about the use of remnant stomach in reconstruction for subtotal esophagectomy subsequent to distal gastrectomy. Several hypotheses are made to explain the maintenance of the gastric vascular integrity as its intramural network without micro-vascular anastomosis.

## Background

In a 10° year period between 1994 and 2004, 126 patients with primary esophageal cancer underwent esophageal resection in our Department of Surgery. Surgical procedures were: 74 (57%) two-phase subtotal oesophagectomy, 30 (23%) transhiatal, 10 (9%) stripping, 10 three-phase subtotal esophagectomy and 2 other procedures (endoscopic resections). After esophageal resection for carcinoma reconstruction of the alimentary tract can be achieved using stomach, colon or jejunum. When technically possible the stomach is the organ of choice, since esophago-gastric anastomosis has been demonstrated to present a lower incidence of complications (i.e. leakages). Colon or jejunum may be used in patients previously undergone to partial gastric resection or total gastrectomy. This is confirmed by our experience: in 112 patients (90%) with primary esophageal cancer, alimentary tract reconstruction was achieved by means of esophago-gastric anastomosis. Reconstruction was performed using colon in 10 (8%) cases and jejunum in 2 (2%). One patient had previously undergone partial gastrectomy and manifested a lower thoracic esophageal carcinoma. Two-phase subtotal esophagectomy was carried out and reconstruction was achieved using the remnant stomach without micro-vascular anastomosis. We wander why gastric remnant in patients who previously received partial gastric resection has not been widely used and if this is a surgical axiom or it is unquestionably demonstrated that it is not technically possible [1,2].

## Case report

**Figure 1:**
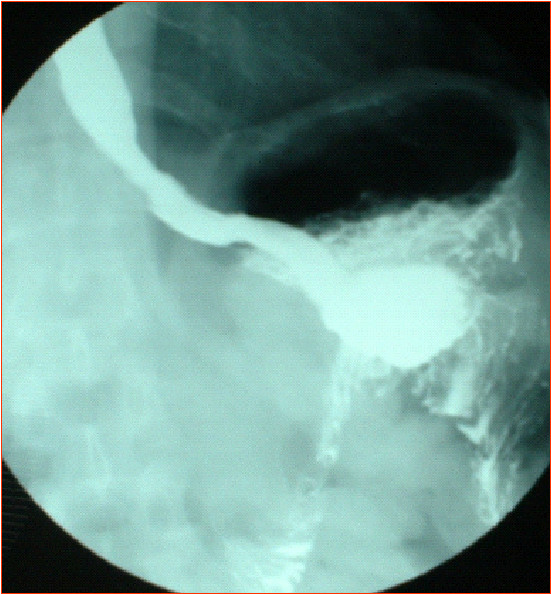
Pre-operative barium meal showing a capacious residual stomach with efficient emptying function through a wide gastrojejunostomy.

A 60-year-old white male was admitted to our Department in January 2003 because of dysphagia and odinophagia. The patient's medical history included perforated benign gastric ulcer treated in 1965 by means of distal gastrectomy and ante colic end to side gastrojejejunostomy (Polya anastomosis). An esophagoscopy confirmed an ulcerated tumour of the lower third esophagus with the upper margin located at 35 cm from dental arcade; a squamous cell carcinoma was identified on biopsies. The interval between previous distal gastrectomy and esophagectomy was 38 years. Barium meal showed a capacious residual stomach with efficient emptying function through a wide gastrojejunostomy. The size of the residual stomach was measured before operation with contrast barium meal x-ray: 8 cm in length at the lesser curvature and 11 cm at the transection of the body (Figure 1). Because of no evidence of metastatic disease and satisfactory general health conditions the patient was scheduled for resection of the oesophageal cancer.

**Figure 2:**
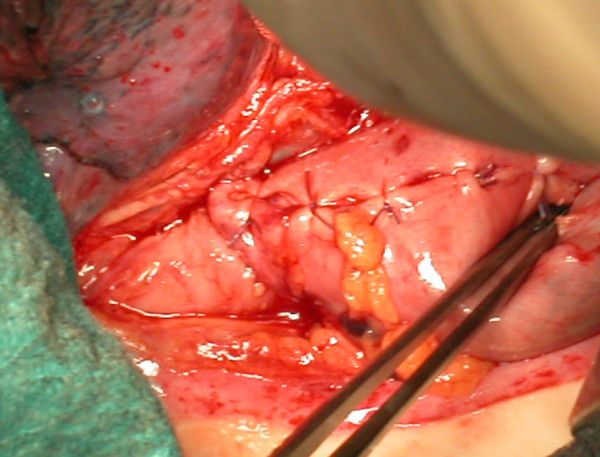
The esophagus is anastomosed to the remnant stomach.

**Figure 3:**
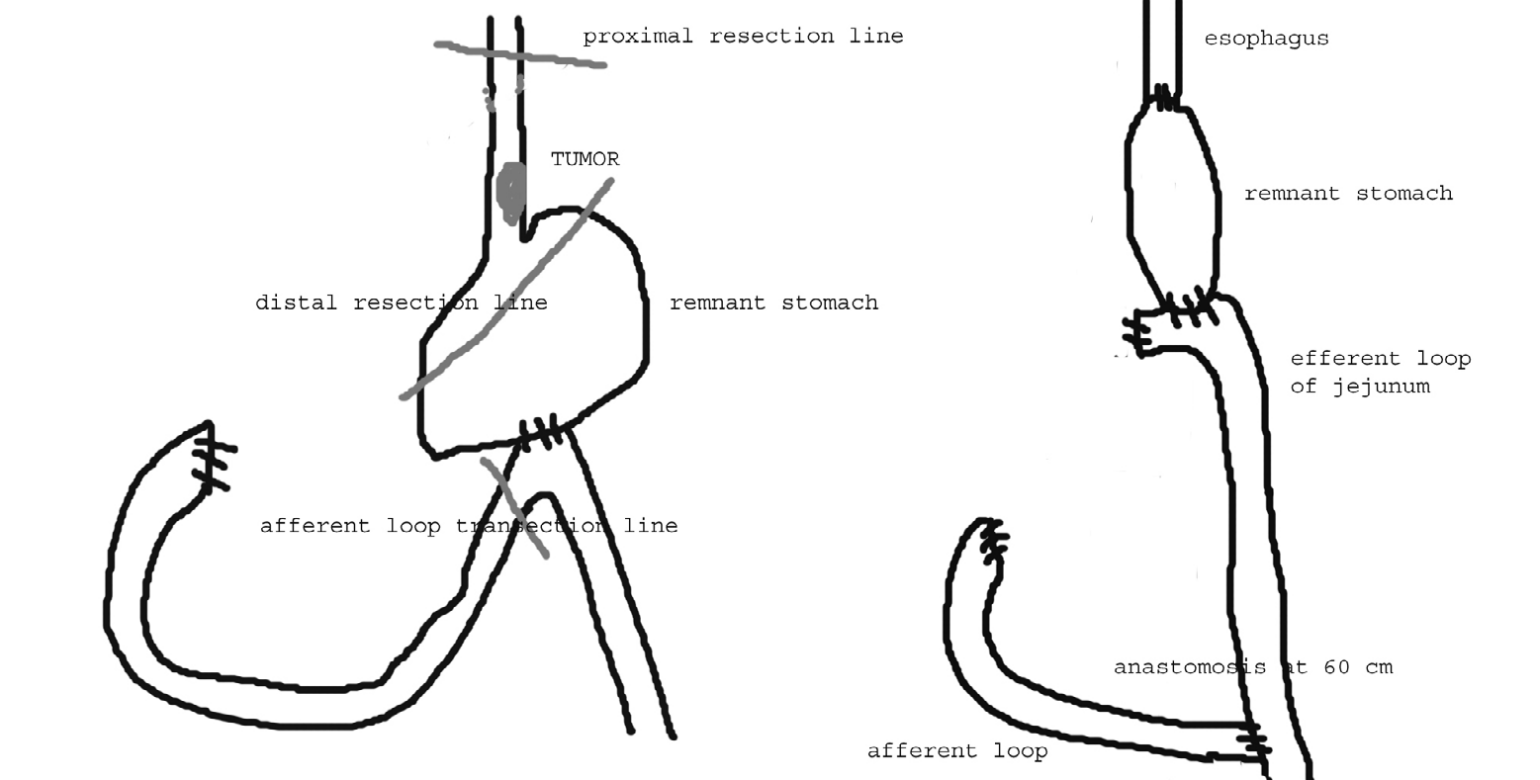
Scheme of the resection area of the remnant stomach and the esophagus (pre-operative situation), and the reconstruction of the organs (post-operative).

Two-phase subtotal esophagectomy via abdominal (bilateral subcostal incision) associated with 5th right thoracotomy with two field lymph node dissection was carried out. In the right upper abdomen the duodenum stump, transverse colon, and lower surface of the liver were tightly adherent. Exploration of the thorax and abdomen excluded presence of distant metastases and confirmed wide gastrojejunal anastomosis. The right gastroepiploic arcade was divided at the time of previous gastrectomy. The residual stomach, afferent and efferent loops of jejunum were fully mobilized from peripheral cohesions. Short gastric vessels were not preserved and the left gastric artery was divided; no jejunal vessel was sacrified. The afferent loop was transected very close to the gastrojejunal anastomosis and the gastric end was closed. The other end was covered with gauze and closed. The prepared stomach roll was pulled up in continuity with the efferent loop through the posterior mediastinal route into the right emithorax. The upper end of the oesophagus was resected 5 cm above the tumour. The specimen was removed after careful sleeve resection of the lesser curve. Proximal and distal resection margins were tested by frozen-section histology assessment. The remnant stomach appeared rosy with adequate bloody supply probably by the efferent jejunal flap with its wide gastrojejunal anastomosis (this was checked by transilluminating the mesenteric vessels). The esophagus was anastomosed mechanically with a circular stapler by an anterior gastrotomy to the high point of the remnant stomach (fundus) at the level of the azygos arcade. No micro vascular anastomosis was performed. At the end there was absence of tension in the anastomosis (Figure 2). The afferent loop was rejoined to the efferent loop at about 60 cm below the original gastrojejunostomy by Roux-en-Y method (Figure 3). A feeding jejejunostomy was made and the abdominal incision was sutured.

Pathological examination revealed a squamous cell tumour of 23 mm in diameter pT3, pN0, M0, R0, G2 (Stage IIA). None coexisting neoplastic lesion was found in the resected esophagus. Evidence of areas of mild dysplasia and esophagitis of the noncancerous esophageal mucosa surrounding the cancer lesion and at the esophagogastric junction was randomly observed. None of the 15 lymph nodes dissected were involved. Postoperative course was uneventful. On follow-up, two years after operation, the patient was alive without evidence of recurrence. The patient had an improved intake of food, mainly managing a semi-solid diet with an average intake per meal was 400–500 mL. Endoscopy findings, barium meal contrast exam and angio-CT scan confirmed good vascularity of the jejunal flap and stomach.

## Conclusion

The relationship between previous gastrectomy and subsequent occurrence of primary malignant esophageal tumour remains controversial. Maeta reported that of 129 patients surgically treated for esophageal cancer 12 (9%) had previously undergone partial gastrectomy [1]. A possible explanation is that the development of esophageal cancer after gastrectomy is related to post-gastrectomy nutritional changes and/or post-operative gastroesophageal reflux [1,2]. Usually the interval between gastrectomy and esophagectomy is shorter in patients who underwent gastrectomy for gastric cancer compared with those who underwent gastrectomy for peptic ulcer [2]. Furthermore, the interval between gastrectomy and development of oesophageal cancer in patients who underwent Billroth I reconstruction is reported to be shorter compared with those who underwent Billroth II reconstruction [2]. Reconstruction after subtotal esophagectomy could represent a serious problem when a previous gastric resection has been performed. Colon or jejunum are more frequently used. Few technical reports have been reported in literature about the use of remnant stomach in reconstruction for subtotal esophagectomy subsequent to distal gastrectomy [3,4]. We presented a case of a patient with distal oesophageal cancer previously treated with partial gastrectomy. Reconstruction has been achieved using the remnant stomach without micro-vascular anastomosis. At the end of the procedure, the macroscopic aspect of the remnant stomach appeared to have an adequate bloody supply; thus no other options such as short segment jejunal interposition or direct Roux-y esophagojejunostomy were performed. Moreover, not infrequently, the marginal artery is found to be insufficient calibre to maintain viability of a transposed colon.

Several *hypothesis *can be made: the reconstituted microvascular supply from the anastomosed efferent jejunal loop with its wide gastrojejunal anastomosis contributed to the maintenance of the gastric vascular integrity as its intramural network. Vascular adaptation is a more likely hypothesis for the adequate blood supply than jejunal vessels: Reavis demonstrated that the delay effect is associated with both vasodilation and angiogenesis and results in increased blood flow to the gastric fundus prior to esophagogastric anastomosis in animals: delayed operations have less anastomotic collagen deposition and ischemic injury than those undergoing immediate resection [5]. Clinical application of the delay effect in patients undergoing esophagogastrectomy may lead to a decreased incidence of leak and stricture formation.

At the third post-operative month, an angio-CT scan, demonstrated good vascularity of the jejunal flap and residual stomach without any vascular congenital abnormality of the aorta and its branches. The prepared stomach roll was pulled up in continuity with the efferent loop with posterior mediastinal route as it provides the shortest distance between abdomen and the thorax. A sufficient long jejunal flap must be needed. Pre-operative and intra-operative assessments revealed the stomach had an adequate length. Previous gastrectomy often causes strict adhesions between the mesocolon and the adjacent organs, making difficult the use of colon for reconstruction. The reduction in number of surgical manoeuvres beneath the transverse colon and few bowel anastomosies represent a real advantage. Chen and Lu proposed the resection of the tumour through left thoracotomy, preserving the left short gastric artery and transporting the residual stomach, the spleen and tail of the pancreas into the left thoracic cavity, and using the residual stomach to reconstruct the alimentary tract and preserve vascular integrity of the stomach [3]. Matsubara proposed micro-vascular anastomosis [4]. There are still some questions: first, a previous gastric procedure with additional nodal dissection might result in changing the pattern of lymph node spreading of a distal oesophageal cancer. Second, it is important to obtain adequate clear margins using the remnant stomach as an oesophageal substitute. The remnant stomach after partial gastrectomy should not be used as esophageal substitute if you pretent to perform cervical anastomosis. There is no data available in literature of the micro-vascularization of the remnant stomach and the role of the efferent stump and the length gastrojejunal anastomosis in maintaining the blood support to the stomach [6]. Periodic surveillance is mandatory in patients who had partial gastrectomy and, if oesophageal cancer is present, its location and stage must be determined.

Finally, in the authors' experience, this technique proved to be efficient with no postoperative complication and long follow-up. The technique must be performed in Institutions with well-trained surgeons and high volume UGI procedures. We recommend further reports to verify the usefulness of the proposed technique.

## Abbreviations

UGI: upper gastrointestinal

CT: computer tomography

## Competing interests

The author(s) declare that they have no competing interests.

## Authors' contributions

GD participated in acquisition of data

FR participated in study conception and design

LB conceived the analysis and interpretation of data

GD and GC carried out drafting of manuscript

RD conceived the critical revision and supervision

All authors read and approved the final manuscript
